# Evaluation of a multi-component training programme for employees aged 50+

**DOI:** 10.1007/s10433-022-00715-0

**Published:** 2022-07-11

**Authors:** Tanja Hüber, Udo Käser, Lena Stahlhofen, Lara Görtner, Una Röhr-Sendlmeier

**Affiliations:** Department of Developmental and Educational Psychology, Rheinische Friedrich-Wilhelms-Universität Bonn (1040), Kaiser-Karl-Ring 9, 53111 Bonn, Germany

**Keywords:** Employees aged 50 +, Multi-component training, Cognitive training, Stress management training, Competence training, Train-the-trainer

## Abstract

Lifelong learning offers an opportunity for mature employees to stay adept in the light of changing demands, to promote health and counteract physical and cognitive decline. This intervention study evaluates the effects of a multi-component training programme for employees aged 50+ , focussing on competence expectations, stress management, cognitive, metacognitive and psychomotoric training. Effects were evaluated in a longitudinal control group design with follow-up after six months (24 training groups, *n* = 247, participants per group: *M* = 13.04, *SD* = 2.44; control group, *n* = 199). To control for experimenter effects the same programme was administered to 6 additional groups by trained instructors (*n* = 54, participants per group: *M* = 11.83, *SD* = 3.37). To validate effects of the multi-component training 12 supplementary groups were included, with 4 groups each focusing on either the competence (*n* = 49, participants per group: *M* = 15.00, *SD* = 0.00) or cognitive (*n* = 43, participants per group: *M* = 14.25, *SD* = 1.50) or stress management components (*n* = 41, participants per group: *M* = 14.50, *SD* = 0.58). Data of 633 adults (mean age: *M* = 55.03, *SD* = 3.71 years) were analysed. Participants reported high acceptance of the programme. The multi-component training programme was effective regarding improvements in subjective health, self-concept of professional competence, self-efficacy, coping with stress and cognitive abilities with long-term effects for the latter four. Trainings administered by trained instructors had similar effects to those administered by the programme’s designers. The single-component trainings led to specific effects in the focused areas, overall comparable to those of the multi-component training. Unexpectedly, cognitive effects were obtained by all single-component trainings. Subjective health and self-efficacy were only promoted by the multi-component training, indicating broader effects. The results are discussed with respect to strengths and limitations of the study, possible mechanisms underlying the effects, suggestions for further research as well as for the training’s implementation in business practice.

## Introduction

Against the background of demographic and economic changes, employers are facing the necessity to maintain their employees’ physical, mental and psychological health, competence and performance which in turn are crucial for the companies’ success (Chuang and Graham 2020). Due to rapidly changing working environments, furthering personal development has become most important (Arnold et al. 2018). Holistic trainings that consider cognitive, physical, social and emotional aspects offer a promising approach to face these demands (Ott and Grotensohn 2014). Learning environments for employees aged 50+ should be designed specifically for this target group (Brünner 2011), since ageing individuals do not necessarily learn less effectively, but rather differently than younger people (Käser and Röhr-Sendlmeier 2002, 2012).

While research on ageing employees has grown over the past decades, there is a lack of specific intervention studies targeting mature employees (Truxillo et al. 2015). Reviews point to only few interventions focusing on improving health, mostly by physical training, imparting knowledge and individual coaching (Poscia et al. 2016; Söderbacka et al. 2020). There are also examples of cognitive and stress management trainings specifically designed for mature employees (e.g. Bundesanstalt für Arbeitsschutz und Arbeitsmedizin 2012). But scientifically designed and evaluated holistic trainings for employees aged 50+ are still lacking.

To ensure the effectiveness of educational programmes, systematic evaluation is essential. Dismantling studies can help understand which elements of a training programme cause specific effects by comparing a full training version with training versions comprising only one or several components (Papa and Follette 2015). Furthermore, experimenter effects are worth investigating to prevent wrong interpretations of training effects. Experimenter effects arise when instructors hold beliefs about the participants or the training, which affect their behaviour unconsciously, so that in turn the participants’ behaviour, emotions and cognition are influenced (Rosenthal and Rosnow 2009).

Considering age-related changes and needs of mature employees, target-group-specific trainings should contain cognitive training, stress management and a reflection of individual competencies. Mature employees often do not value their individual competencies, although these can provide resources in dealing with age-related changes and challenges at work (Staudinger and Bowen 2011). While some studies do not find differences in the general level of experienced work-related stress between younger and mature employees (Rauschenbach et al. 2013), others document higher levels of stress for the latter regarding pace of work and fear of not being able to keep up (Techniker Krankenkasse 2016). Chronic distress is related to poor health, may lead to absence from work and impair (cognitive) performance (Phillips 2017; Techniker Krankenkasse 2016). Although a formerly prevailing deficit model of ageing is no longer accepted, some losses have to be accounted for, e.g. in sensomotor functions or fluid intelligence. In work contexts, fluid intelligence—including working memory and mental speed—is highly relevant, especially concerning information analysis, decision-making and handling unpredictable situations (Gajewski and Falkenstein 2011).

Age-related losses regarding basic cognitive abilities like mental speed and concentration can be compensated for by training (Kelly et al. 2014). Not only cognitive training interventions (Rebok et al. 2014), but also intellectual engagement and cognitively demanding (leisure) activities (Phillips 2017) are essential for maintaining and promoting cognitive functioning, especially fluid intelligence. Beneficial effects of playing challenging strategic games on cognitive abilities, like attention and mental speed, have been shown in different studies, e.g. for chess and the Asian game Go (Aciego et al. 2012; Iizuka et al. 2019a).

Metacognitive elements, like monitoring cognitive processes and using strategies (Bailey et al. 2010), can support cognitive trainings’ effectiveness by improving problem-solving and supporting transfer to untrained tasks (O’Connell and Robertson 2012). Metacognitive trainings have been shown to be effective in counteracting age-related memory decline in late adulthood (Hertzog and Dunlosky 2011). Using cognitive strategies is not only associated with memory, but also with everyday functioning (Gross and Rebok 2011).

Also, physical activity can counteract age-related declines in volume, functioning and connectivity of brain areas associated with cognitive abilities, e.g. by stimulating cerebral metabolism and neural plasticity (Kramer and Colcombe 2018). Positive effects on cognitive abilities have been shown for dancing, fitness-, coordination- and balance-training (Kramer and Colcombe 2018; Predovan et al. 2019; Voelcker-Rehage et al. 2010, 2011) with combined physical and cognitive training often being more effective than either alone (Joubert and Chainay 2018; Oswald et al. 2006).

Physical activity also helps to reduce and prevent stress, e.g. by reducing the physiological stress response (Phillips 2017). Stress occurs when the demands a person faces exceed the subjective resources available to meet them (Lazarus and Lazarus 1994). Thus, the experienced level of stress is dependent on individual resources and coping strategies (Sonnentag and Frese 2003). Stress management trainings focussing on enhancing the latter have shown to be effective, e.g. regarding the reduction in stress-related symptoms (Richardson and Rothstein 2008).

Relaxation techniques, like Progressive Muscle Relaxation (PMR), are used for preventing stress-related disorders or optimizing performance. They can support effects of stress management and cognitive trainings, e.g. by reducing the experienced level of stress, having a regenerative effect after cognitive load and fostering concentration, self-regulation and well-being (Holman et al. 2018; Petermann and Vaitl 2014).

Competence, including social, personal, methodological and vocational competencies (Wottreng 1999), is acquired continuously. Concomitantly, the self-concept is adapted to different environments and challenges (Brandtstädter 2010). Handling age-related changes appropriately is central for maintaining individual functioning, positive self-concept and quality of life. According to Baltes and Baltes (1990), strategies of resource-oriented goal selection, optimal use of resources and compensation of impaired abilities are important for minimizing losses and maximizing gains. A systematic reflection of one’s experiences in different contexts can help become aware of one’s multifaceted competencies and resources (German Institute for Adult Education 2016). This, in turn, can have a positive impact on self-concept and self-efficacy.

Gerontological research shows that loss of competencies is not inevitable and individual competencies can be maintained or even increased by training during working life and until old age (Zacher 2015). Against this background, a multi-component group intervention for employees aged 50+ was developed. It aims at maintaining and promoting individual resources and abilities that are important at work and in private life—self-concept of professional competence, coping with stress and cognitive abilities. Thus, it aims at improving health and self-efficacy. A group training was chosen to take advantage of participants’ interactions while dealing with training contents and because individual trainings are unlikely to be implemented in companies. Our research questions, developed successively in the course of the project, were: (1) Does the multi-component training produce short-term and long-term effects? (2) To control for experimenter effects: Are training effects independent of instructors? (3) To gain further insight into the components evoking the results: Can differential effects of single-component trainings be found? 4) To examine possible synergy effects evoked by the combination of modules: Are effects of the multi-component training different from those of single-component trainings?

## Methods

### Study design

The effectiveness of a holistic training programme was evaluated in an intervention study from 2013 until 2019 using different methodological approaches: First, effects of the multi-component training were tested in an intervention group (IG_mult_) against a control group (CG) in a longitudinal design and with a follow-up after six months in IG_mult_. Second, training effects obtained due to developers’ bias were controlled for by additional courses administered by trained instructors (train-the-trainer—TTT) who had not been involved in the development of the training programme (IG_TTT_). Third, to test for differential effects of the multi-component training, three single-component interventions (competence: IG_comp_; stress management: IG_stress_; cognition: IG_cogn_) were conducted.

Due to organizational reasons regarding data collection and training implementation, assessors could not be held blind to participant intervention. Randomization was limited in three ways: First, companies decided whether they wanted to take part in the study. Second, within companies it was necessary to allow for interest-driven choice of training participation, as trainings were offered during work time. Third, participants could not be assigned randomly across training groups, because trainings took place in different companies.

Questionnaires and tests were administered by trained staff, using standardized manuals, during the first (t1) and last sessions (t2) of the trainings and about six months after the trainings (t3, intervention groups). CG was tested during two appointments (t1, t2) with an interval of approximately four months (equivalent to the intervention period).

### Sample

Participants were mainly recruited in North Rhine-Westphalia, Germany, by informing various companies about the project, via email and local newspapers or in person. The target group were working people aged 50+, who received information about the intervention and participated voluntarily. The guidelines for ethical research of the 1964 Helsinki declaration and its later amendments were abided by throughout the research process. The study design was discussed with the chairman of the ethics committee of the Institute of Psychology, Bonn University. Neither then nor at any point of the study was any objection raised. The participants were informed about the study’s scientific purpose including publication, and their consent was registered before data collection. The intervention was conducted in companies of various economic sectors (e.g. insurance business, manufacturing industries, education) or in groups of people from different companies. The multi-component training was administered to 24 groups, single-component trainings to 12 groups (4 groups for each version) and TTT-trainings to 6 groups (from the same economic sectors), with an overall average group size of 13.31 participants (*SD* = 2.40, range 6 to 16). Controls did not receive any treatment and continued their normal working routine. Including an active CG was not possible, because companies did not agree on releasing their staff for alternative activities during work time. Companies also did not agree to testing the CG at t3 during working hours.

**Fig. 1:**
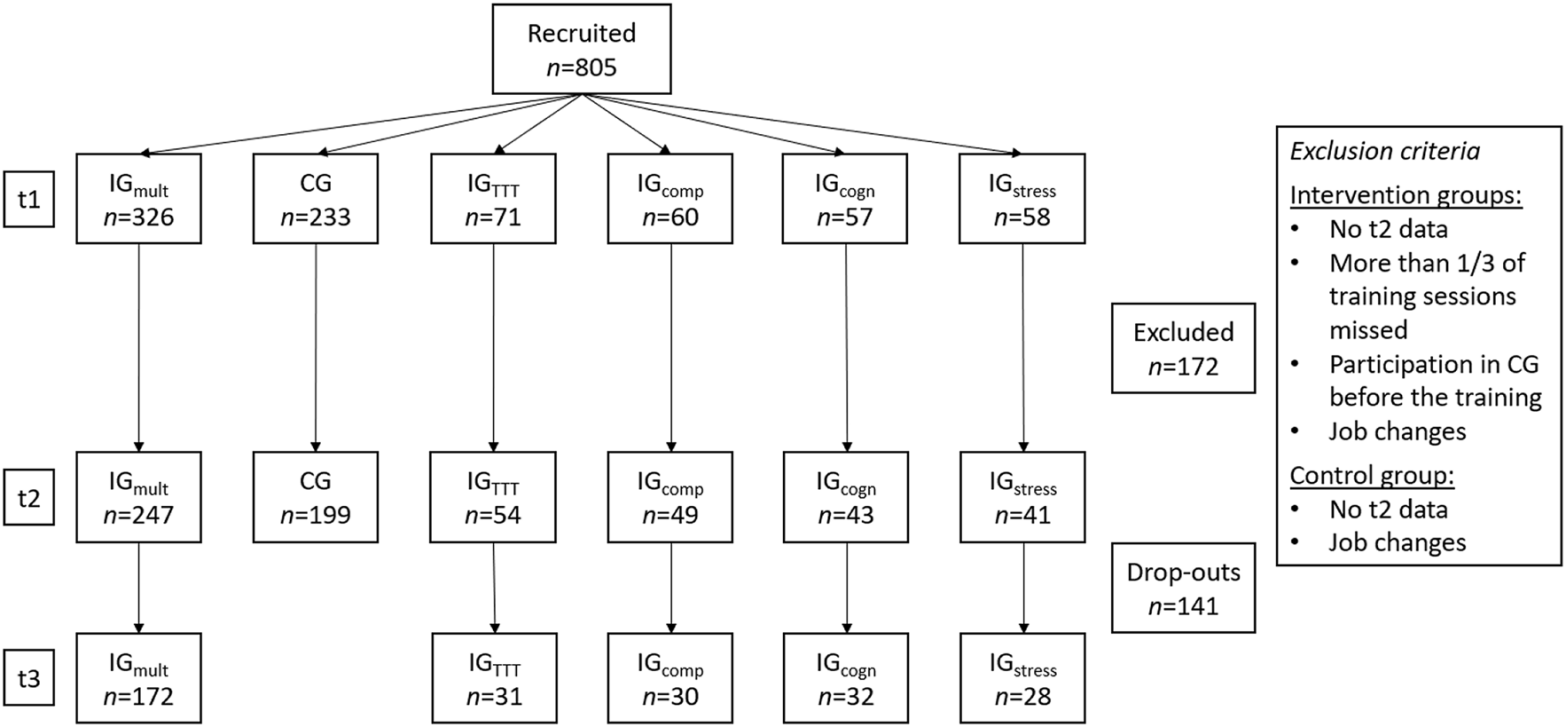
Flowchart illustrating the number of participants and exclusion criteria

Figure 1 shows the number of participants across all groups for pre-, post- and follow-up-measurements and the exclusion criteria. The drop-outs did not differ from the remaining sample in gender and education, but their average age (*M* = 56.09, *SD* = 4.42) was significantly higher than in the remaining sample (*M* = 55.04, *SD* = 3.71, *p* = 0.006, *d* = 0.244). Complete data sets (t1 and t2) were available from 633 individuals (433 women, 68.40%). Four hundred and thirty eight participants (69.30%) held a qualification for entering university or college. Differences between IG_mult_ and the other groups (Table 1) are of minor relevance since testing for associations of sample characteristics with gains in the outcome variables in general showed no significance.

**Table 1:** Sample characteristics

Characteristics	IG_mult_	IG_TTT_	IG_comp_	IG_cogn_	IG_stress_	CG
Sample size, *n*	247	54	49	43	41	199
Sex, females, *n* (%)	168 (68.02)	43 (79.63)*	41 (83.67)*	29 (67.44)	32 (78.05)	120 (60.30)*
Age (years), *M* (*SD*)	55.01 (3.37)	53.87 (3.46)*	54.71 (3.85)	56.26 (4.26)*	54.95 (3.21)	55.23 (4.02)
Educational level, (Fach-) Abitur^a^, *n* (%)	162 (65.59)	39 (72.22)	39 (79.59)*	32 (74.42)	36 (87.80)*	130 (65.33)

^a^German qualification for entering university or college

*Significant difference compared to IG_mult_ (*p* < .10)

### Intervention

Training groups of up to 16 participants were instructed by two trainers using a standardized manual containing psychoeducational explanations and extensive practice elements. The training included short lectures, individual reflection, group discussions and different types of written and activating exercises. Most physical exercises allowed for variation, accounting for individual differences and promoting optimal training at medium difficulty. By discussing strategies and exchanging experiences, participants were encouraged to integrate exercises and new behaviour into everyday life.

### Training versions

The multi-component version, comprising 15 weekly sessions of 2 and a half hours, contained five modules: (1) competence, (2) stress management, (3) cognition, (4) metacognition and (5) psychomotricity (for details see Röhr-Sendlmeier et al. 2019). The single-component versions, comprising 7 weekly sessions of 2 h, focused on specific aspects of the multi-component version: (1) competence, (2) stress management, or (3) cognition and metacognition, each combined with activating exercises of the psychomotoric training. This was motivated by findings of Oswald et al. (2006) showing that combined trainings, especially with psychomotoric elements, proved to be most effective. Single-component versions contained the identical content of the respective module of the multi-component training, so that the actual time spent on dealing with the respective topics was the same, with the psychomotoric activation filling additional time in each single-component version.

The TTT-programme, comprising 60 h, aimed at ensuring that psychology students and employees of cooperating companies who later took on the role of trained instructors acquired sufficient psychological background knowledge and extensive practical experience as trainers. After completing the TTT-programme, trained instructors administered the multi-component version in pairs.

### Modules

*Competence training* aimed at facilitating perception, appreciation and use of individual abilities. Its key component was systematic reflection on and documentation of subjective experiences in different occupational and private contexts, followed by deducing competencies that helped to cope with these situations (German Institute for Adult Education 2016).

*Stress management training* intended to reduce physical tension and inner restlessness and to improve coping with stress, focusing on individual resources and strategies. Key components were reflecting on individual stressors and stress reactions, re-evaluation of personal attitudes reinforcing experience of stress and developing coping strategies like stopping rumination, setting priorities and planning recovery time (Hillert et al. 2012; Kaluza 2018).

*Cognitive training* included learning basic rules and strategies of the strategic game Go, widely unknown in Germany, solving complex problems on exercise sheets and playing the game (Dickfeld 2016; Digulla et al. 2008).

*Metacognitive training* contained information about functioning of memory, impacts on memory performance and memory strategies as well as practical exercises (Croisile 2011). The aforementioned modules also implied metacognitive elements (e.g. cognitive strategies and monitoring processes, metacognitive control).

*Psychomotoric training* targeted activation by practicing short and age-appropriate exercises like balance and coordination exercises (Beigel 2015; Schmidt et al. 2013) and relaxation, using the PMR technique originally introduced by Jacobson (1929). PMR aims at perceiving and reducing physical tension and intensifying relaxation by inducing a change between tension and subsequent relaxation of distinct muscle groups.

### Outcome measures

Training effects were calculated based on self-ratings, using questionnaires with a five-point rating scale from *1* = *disagree completely* to *5* = *agree completely*, and psychometric cognitive tests. All scales were standardized psychological questionnaires, partly shortened for reasons of economic assessment. Reliabilities were calculated on the basis of the complete sample (*n* = 633) and showed to be very satisfactory in spite of reduced item numbers (Table 2). Subjective health was measured by a single item (“How is your health in general?”) using a five-point rating scale from *1* = *very good* to *5* = *very bad*. Demographic data were assessed at pre-test regarding sex, age and educational level by single items. At post-test, participants were asked whether they would recommend the training.

**Table 2:** Outcome measures

Scale	Questionnaire/psychometric test	No. of items	Reliability (Cronbach's Alpha)	Test score
*Self-efficacy*				
Self-efficacy	Allgemeine Selbstwirksamkeit Kurzskala (ASKU; Beierlein et al. 2012)	3	.809	Arithmetic mean (1 to 5)
*Self-concept of professional competence*				
Expertise	Scale for measuring professional expertise (Goossens 2013)	5	.703	Arithmetic mean (1 to 5)
Vocational competencies	Fragebogen zur Selbsteinschätzung beruflicher Kompetenz (SBK; Sonntag and Schäfer-Rauser 1993)	5	.746	Arithmetic mean (1 to 5)
Methodological competencies	"	5	.834	"
Social competencies	"	6	.716	"
Motivation on the job	Bochumer Inventar zur berufsbezogenen Persönlich-keitsbeschreibung (BIP; Hossiep and Paschen 2003)	11	.825	"
Flexibility on the job	"	14	.863	"
Affective commitment to the organization	Commitment-Skalen (COMMIT; Felfe and Franke 2012)	5	.874	"
*Coping with stress*				
Subjective importance of work	Arbeitsbezogenes Verhaltens- und Erlebensmuster (AVEM; Schaarschmidt and Fischer 2008)	4	.800	stanine values
Professional ambition	"	4	.778	"
Willingness to work to exhaustion	"	4	.838	"
Striving for perfection	"	4	.715	"
Distancing ability	"	4	.862	"
Resignation tendency when facing failure	"	4	.755	"
Proactive problem solving	"	4	.811	"
Inner calm	"	4	.826	"
Successful experiences at work	"	4	.811	"
Satisfaction with life	"	4	.842	"
Experience of social support	"	4	.796	"
*Cognitive abilities*				
Mental speed	Zahlen-Verbindungs-Test (ZVT; Oswald and Roth 1987)	4	.914	Arithmetic mean (All sets)
Concentration	Alters-Konzentrations-Test (AKT; Gatterer 1990)	3	.816	"

According to the content of the respective training, participants of the intervention groups received feedback after pre- and post-tests on cognitive performance, stress management, competencies and self-efficacy. Participants of CG received feedback on cognitive performance, stress management and self-efficacy only after post-tests to avoid changes in normal behaviour induced by test results.

### Statistical analysis

For statistical analyses IBM SPSS 26 was used. *Χ*^2^-tests, independent samples *t*-tests, paired sample *t*-tests and mixed-design ANOVAs with repeated-measures factor time (2: t1 vs. t2) and between-subjects factor group (2: IG_mult_ vs. CG) were conducted to examine research questions 1 and 3. Violations of assumptions were considered when testing for statistical significance. *α* ≤ 0.05 was considered to indicate statistical significance (respectively, *α* ≤ 0.10 for comparing groups at baseline). Effect sizes are reported as Cohen’s *d*, with *d* ≥ 0.2 indicating small, *d* ≥ 0.5 indicating medium and *d* ≥ 0.8 indicating large effects (Cohen 1988). They were calculated by the formula suggested by Dunlap et al. (1996), yielding conservative measures to ensure caution in interpreting results. To answer research questions 2 and 4, effect sizes of intervention groups (IG_mult_ vs. IG_TTT,_ IG_mult_ vs. single-component versions) were computed for variables with a significant interaction of group and time when comparing IG_mult_ and CG or near to small effects of this interaction. These were compared using Psychometrica (Lenhard and Lenhard 2016, https://www.psychometrica.de/effect_size.html) by transforming Cohen’s *d*, resulting from paired sample *t*-tests, into Pearson product-moment correlation coefficient *r.* Subsequently Cohen’s *q*, representing the difference between Fisher z-transformations of two correlations *r*, was calculated with *q* ≥ 0.1 indicating small, *q* ≥ 0.3 indicating medium and *q* ≥ 0.5 indicating large effects (Cohen 1988).

## Results

### Acceptance of training

Totally, 95.49% of participants would recommend the training (IG_mult_: 97.06%, IG_TTT_: 92.45%, IG_comp_: 87.23%, IG_stress_: 95.12%, IG_cogn_: 100.00%). Recommendation only differed significantly between IG_mult_ and IG_comp_ (*p* = 0.003, *d* = 0.355).

### Training effects

Table 3 gives the results of interactions between groups IG_mult_ and CG and time. Effects of significant results were small, unless stated otherwise.

**Table 3:** Interaction between group and time based on ANOVAs (IG_mult_: *n* = 247, CG: *n* = 199)

Scale	t1 *M* (*SD*)	t2 *M* (*SD*)	*d* (CI-95%)	*p*
*Subjective health*				
IG_mult_	2.21 (0.68)	2.12 (0.71)	0.277 (0.09–0.47)	**
CG	2.00 (0.64)	2.06 (0.64)		
*Self-efficacy*				
IG_mult_	4.01 (0.50)	4.15 (0.54)	0.223	*
CG	4.10 (0.52)	4.13 (0.51)	(0.04–0.41)	
*Self-concept of professional competence*				
Expertise				
IG_mult_	4.02 (0.54)	4.16 (0.53)	0.353	***
CG	4.16 (0.50)	4.15 (0.46)	(0.16–0.54)	
Vocational competencies				
IG_mult_	4.17 (0.51)	4.24 (0.46)	0.124	
CG	4.13 (0.44)	4.15 (0.45)	(−0.06–0.31)	
Methodological competencies				
IG_mult_	3.96 (0.63)	4.03 (0.59)	0.254	**
CG	3.91 (0.63)	3.87 (0.62)	(0.07–0.44)	
Social competencies				
IG_mult_	4.27 (0.47)	4.27 (0.46)	0.049	
CG	4.23 (0.44)	4.21 (0.46)	(−0.14–0.24)	
Motivation on the job				
IG_mult_	2.88 (0.60)	2.87 (0.57)	0.017	
CG	2.90 (0.62)	2.89 (0.67)	(−0.17–0.20)	
Flexibility on the job				
IG_mult_	3.29 (0.55)	3.34 (0.54)	0.176	
CG	3.27 (0.64)	3.25 (0.62)	(−0.01–0.36)	
Affective commitment				
IG_mult_	3.77 (0.86)	3.82 (0.82)	0.264	**
CG	3.87 (0.81)	3.79 (0.85)	(0.08–0.45)	
*Coping with stress*				
Subjective importance of work				
IG_mult_	3.58 (1.77)	3.45 (1.61)	−0.087	
CG	3.84 (1.65)	3.83 (1.88)	(−0.27–0.10)	
Professional ambition				
IG_mult_	4.20 (1.73)	4.38 (1.61)	0.116	
CG	4.48 (1.71)	4.53 (1.83)	(−0.07–0.30)	
Willingness to work to exhaustion				
IG_mult_	3.93 (2.00)	3.68 (2.06)	0.208	*
CG	3.64 (2.13)	3.68 (2.13)	(0.02–0.40)	
Striving for perfection				
IG_mult_	4.36 (1.69)	4.25 (1.84)	0.073	
CG	4.01 (1.76)	4.01 (1.86)	(−0.11–0.26)	
Distancing ability				
IG_mult_	5.77 (1.81)	6.23 (1.82)	0.279	**
CG	5.93 (1.90)	6.07 (1.88)	(0.09–0.46)	
Resignation tendency				
IG_mult_	5.09 (1.91)	4.79 (1.76)	0.165	
CG	4.62 (1.75)	4.55 (1.69)	(−0.02–0.35)	
Proactive problem solving				
IG_mult_	4.36 (1.83)	4.66 (1.83)	0.072	
CG	4.62 (1.83)	4.83 (1.68)	(−0.12–0.26)	
Inner calm				
IG_mult_	4.65 (1.95)	5.19 (1.95)	0.313	**
CG	5.23 (1.93)	5.37 (1.89)	(0.13–0.50)	
Successful experiences at work				
IG_mult_	5.09 (1.97)	5.38 (1.92)	0.133	
CG	5.06 (1.86)	5.17 (1.96)	(−0.06–0.32)	
Satisfaction with life				
IG_mult_	5.47 (2.07)	5.71 (2.01)	0.066	
CG	5.37 (1.89)	5.51 (1.98)	(−0.12–0.25)	
Experience of social support				
IG_mult_	4.74 (1.80)	4.72 (1.77)	0.043	
CG	4.85 (1.74)	4.78 (1.66)	(−0.15–0.23)	
*Cognitive abilities*				
Mental speed				
IG_mult_	2.38 (0.46)	2.56 (0.47)	0.501	***
CG	2.39 (0.45)	2.43 (0.47)	(0.30–0.68)	
Concentration				
IG_mult_	60.54 (14.29)	68.02 (15.36)	0.242	*
CG	62.16 (12.32)	66.82 (12.66)	(0.05–0.43)	

*p < .05; **p < .01; ***p < .001

Comparing subjective health before and after training, IG_mult_ improved significantly more than CG (see Table 3). Additional *t*-tests revealed that at t1 health status was rated lower in IG_mult_ (*p* = 0.001, *d* = 0.329) than in CG; at t2 there was no significant difference between IG_mult_ and CG (*p* = 0.396).

For self-efficacy, time and group also interacted significantly in favour of IG_mult_ (see Table 3). While at t1 self-efficacy was descriptively lower in IG_mult_ than in CG, it was slightly higher in IG_mult_ than in CG at t2 due to significant improvement in IG_mult_ from t1 to t2 (see Table 4).

**Table 4:** Results of *t*-tests comparing intra-group developments from t1 to t2 and t1 to t3 and comparison of effect sizes (Cohen’s *q*) between IG_mult_ and IG_TTT_, IG_comp_, IG_cogn_, IG_stress_ for both intervals

Scale	t1 *M* (*SD*)	t2 *M* (*SD*)	*p*	Cohen’s *d* (CI-95%)	Cohen’s *q*	t1 *M* (*SD*)	t3 *M* (*SD*)	*p*	Cohen’s *d* (CI-95%)	Cohen’s *q*
*Subjective health*										
IG_mult_	2.21 (0.68)	2.12 (0.71)	**	0.142 (0.04–0.24)		2.23 (0.67)	2.19 (0.74)		0.066 (−0.08–0.21)	
IG_TTT_	2.14 (0.75)	1.92 (0.55)	*	0.323 (0.04–0.60)	0.090	2.16 (0.78)	1.90 (0.47)		0.391 (−0.05–0.82)	0.161
IG_comp_	1.77 (0.63)	1.79 (0.54)		−0.035 (−0.25–0.18)	0.088	1.77 (0.57)	1.83 (0.59)		0.115 (−0.22–0.45)	0.090
IG_cogn_	2.03 (0.54)	1.97 (0.54)		0.095 (−0.14–0.33)	0.023	2.03 (0.59)	2.19 (0.74)		0.233 (−0.27–0.73)	0.149
IG_stress_	2.24 (0.71)	2.21 (0.66)		0.038 (−0.27–0.34)	0.052	2.12 (0.71)	2.04 (0.60)		0.115 (−0.23–0.46)	0.024
CG	2.00 (0.64)	2.06 (0.64)		0.094 (−0.04–0.22)						
*Self-efficacy*										
IG_mult_	4.01 (0.50)	4.15 (0.54)	***	0.276 (0.15–0.41)		4.01 (0.47)	4.09 (0.55)	*	0.162 (0.02–0.30)	
IG_TTT_	3.98 (0.55)	4.11 (0.56)	*	0.245 (0.02–0.47)	0.015	3.89 (0.52)	4.23 (0.57)	**	0.618 (0.23–1.00)	0.223
IG_comp_	3.95 (0.74)	4.04 (0.66)		0.127 (−0.09–0.34)	0.074	3.97 (0.61)	4.08 (0.66)		0.175 (−0.20–0.55)	0.007
IG_cogn_	3.85 (0.49)	3.97 (0.45)		0.245 (−0.02–0.51)	0.015	3.82 (0.47)	3.85 (0.42)		0.069 (−0.20–0.34)	0.046
IG_stress_	3.95 (0.69)	4.03 (0.63)		0.113 (−0.19–0.41)	0.081	3.99 (0.75)	4.02 (0.51)		0.055 (−0.32–0.43)	0.053
CG	4.10 (0.52)	4.13 (0.51)		0.065 (−0.06–0.19)						
*Self-concept of professional competence*										
Expertise										
IG_mult_	4.02 (0.54)	4.16 (0.53)	***	0.249 (0.14–0.35)		4.03 (0.51)	4.13 (0.49)	**	0.206 (0.07–0.35)	
IG_TTT_	4.01 (0.50)	4.10 (0.45)	*	0.201 (0.00–0.40)	0.024	3.97 (0.43)	4.12 (0.46)		0.331 (−0.04–0.70)	0.062
IG_comp_	4.03 (0.63)	4.20 (0.55)	**	0.285 (0.11–0.46)	0.018	3.95 (0.67)	4.14 (0.62)	*	0.288 (0.01–0.56)	0.041
IG_cogn_	4.04 (0.58)	4.04 (0.52)		0.000 (−0.20–0.20)	0.124	4.01 (0.55)	4.01 (0.45)		0.012 (−0.23–0.25)	0.097
IG_stress_	3.83 (0.54)	3.95 (0.54)		0.222 (−0.06–0.50)	0.013	3.87 (0.56)	3.96 (0.45)		0.169 (−0.13–0.47)	0.018
CG	4.16 (0.50)	4.15 (0.46)		0.022 (−0.09–0.13)						
Methodological competencies										
IG_mult_	3.96 (0.63)	4.03 (0.59)	*	0.112 (0.03–0.20)		3.94 (0.62)	3.95 (0.56)		0.022 (−0.10–0.14)	
IG_TTT_	3.89 (0.69)	3.98 (0.62)		0.145 (−0.03–0.32)	0.016	3.84 (0.73)	3.90 (0.63)		0.087 (−0.18–0.35)	0.033
IG_comp_	3.73 (0.85)	3.96 (0.76)	**	0.290 (0.09–0.49)	0.089	3.79 (0.84)	3.95 (0.79)	*	0.204 (0.00–0.41)	0.091
IG_cogn_	3.80 (0.62)	3.87 (0.67)		0.097 (−0.09–0.28)	0.008	3.78 (0.66)	3.79 (0.59)		0.020 (−0.19–0.24)	0.001
IG_stress_	3.79 (0.74)	3.82 (0.69)		0.042 (−0.14–0.23)	0.035	3.78 (0.79)	3.88 (0.74)		0.135 (−0.14–0.41)	0.056
CG	3.91 (0.63)	3.87 (0.62)		0.055 (−0.03–0.14)						
Flexibility on the job										
IG_mult_	3.29 (0.55)	3.34 (0.54)		0.089 (0.00–0.18)		3.29 (0.54)	3.35 (0.55)	*	0.115 (0.02–0.21)	
IG_TTT_	3.16 (0.56)	3.29 (0.54)	*	0.231 (0.05–0.42)	0.071	3.14 (0.54)	3.10 (0.49)		0.069 (−0.23–0.37)	0.092
IG_comp_	3.35 (0.69)	3.48 (0.65)	*	0.192 (0.04–0.34)	0.051	3.27 (0.64)	3.40 (0.61)		0.212 (−0.03–0.46)	0.048
IG_cogn_	3.32 (0.53)	3.37 (0.49)		0.097 (−0.07–0.27)	0.004	3.28 (0.56)	3.34 (0.54)		0.109 (−0.10–0.32)	0.003
IG_stress_	3.02 (0.60)	3.12 (0.62)		0.154 (−0.03–0.34)	0.032	3.06 (0.60)	3.14 (0.74)		0.117 (−0.14–0.37)	0.001
CG	3.27 (0.64)	3.25 (0.62)		0.029 (−0.05–0.11)						
Affective commitment										
IG_mult_	3.77 (0.86)	3.82 (0.82)		0.068 (−0.01–0.14)		3.73 (0.85)	3.77 (0.84)		0.038 (−0.06–0.14)	
IG_TTT_	3.67 (1.00)	3.72 (0.86)		0.054 (−0.11–0.22)	0.007	3.73 (1.01)	3.79 (0.91)		0.061 (−0.12–0.24)	0.012
IG_comp_	3.39 (0.98)	3.42 (1.01)		0.024 (−0.21–0.26)	0.022	3.46 (1.00)	3.56 (0.94)		0.107 (−0.25–0.47)	0.034
IG_cogn_	3.75 (0.82)	3.73 (0.78)		−0.032 (−0.21–0.15)	0.050	3.66 (0.85)	3.68 (0.82)		0.015 (−0.27–0.30)	0.012
IG_stress_	3.67 (0.94)	3.50 (0.88)		0.183 (−0.04–0.41)	0.125	3.73 (0.86)	3.58 (0.74)		0.183 (−0.17–0.53)	0.110
CG	3.87 (0.81)	3.79 (0.85)	*	0.098 (0.00–0.19)						
*Coping with stress*										
Willingness to work to exhaustion										
IG_mult_	3.93 (2.00)	3.68 (2.06)	**	0.123 (0.03–0.21)		3.84 (2.00)	3.35 (1.92)	***	0.249 (0.13–0.36)	
IG_TTT_	3.57 (1.80)	3.13 (1.84)	*	0.244 (0.02–0.47)	0.060	3.47 (1.81)	3.07 (1.64)		0.230 (−0.04–0.50)	0.009
IG_comp_	4.02 (2.18)	4.17 (1.87)		−0.070 (−0.27–0.13)	0.096	4.30 (2.02)	3.97 (1.94)		0.168 (−0.10–0.43)	0.040
IG_cogn_	3.17 (1.91)	3.17 (2.04)		0.000 (−0.17–0.17)	0.061	3.06 (1.79)	3.00 (1.78)		0.035 (−0.19–0.26)	0.107
IG_stress_	5.10 (2.43)	4.63 (2.31)		0.200 (−0.05–0.45)	0.038	4.93 (2.48)	4.07 (2.48)	*	0.344 (0.03–0.66)	0.047
CG	3.64 (2.13)	3.68 (2.13)		−0.019 (−0.11–0.07)						
Distancing ability										
IG_mult_	5.77 (1.81)	6.23 (1.82)	***	0.255 (0.16–0.35)		5.87 (1.72)	6.35 (1.84)	***	0.273 (0.16–0.38)	
IG_TTT_	6.33 (1.66)	6.91 (1.58)	**	0.354 (0.15–0.56)	0.049	6.30 (1.74)	7.00 (1.62)	**	0.414 (0.13–0.70)	0.070
IG_comp_	5.42 (1.89)	5.35 (1.73)		0.034 (−0.17–0.24)	0.144	5.33 (1.47)	5.47 (1.63)		0.085 (−0.21–0.38)	0.094
IG_cogn_	5.90 (1.76)	6.02 (1.81)		0.068 (−0.09–0.22)	0.093	5.91 (1.86)	6.09 (1.94)		0.099 (−0.16–0.36)	0.087
IG_stress_	4.80 (2.05)	5.50 (1.96)	**	0.348 (0.12–0.57)	0.046	5.26 (2.16)	5.48 (1.65)		0.112 (−0.23–0.46)	0.080
CG	5.93 (1.90)	6.07 (1.88)		0.072 (−0.01–0.15)						
Resignation tendency										
IG_mult_	5.09 (1.91)	4.79 (1.76)	**	0.163 (0.07–0.26)		5.12 (1.85)	4.53 (1.85)	***	0.318 (0.19–0.44)	
IG_TTT_	4.80 (1.76)	4.33 (1.84)	*	0.257 (0.00–0.51)	0.047	4.80 (1.95)	3.73 (1.76)	**	0.572 (0.18–0.96)	0.124
IG_comp_	5.44 (2.13)	4.83 (1.74)	*	0.304 (0.06–0.55)	0.070	5.57 (1.70)	5.20 (1.79)		0.210 (−0.15–0.57)	0.054
IG_cogn_	4.88 (1.99)	4.85 (1.78)		0.013 (−0.19–0.22)	0.075	4.88 (2.11)	4.91 (1.87)		0.015 (−0.18–0.21)	0.166
IG_stress_	6.00 (1.65)	5.25 (1.71)	**	0.447 (0.14–0.76)	0.140	5.85 (1.83)	5.07 (1.73)	*	0.436 (0.02–0.84)	0.058
CG	4.62 (1.75)	4.55 (1.69)		0.041 (−0.07–0.15)						
Inner calm										
IG_mult_	4.65 (1.95)	5.19 (1.95)	***	0.275 (0.19–0.36)		4.75 (2.01)	5.28 (2.14)	***	0.254 (0.15–0.36)	
IG_TTT_	4.43 (2.02)	5.09 (1.92)	**	0.338 (0.14–0.54)	0.031	4.27 (2.00)	5.07 (1.96)	**	0.404 (0.11–0.70)	0.074
IG_comp_	4.52 (2.01)	5.02 (1.95)	**	0.252 (0.09–0.42)	0.011	4.23 (1.96)	4.47 (2.10)		0.114 (−0.07–0.30)	0.070
IG_cogn_	4.49 (1.99)	4.80 (1.99)		0.159 (−0.04–0.36)	0.058	4.50 (2.00)	4.66 (2.01)		0.078 (−0.10–0.26)	0.088
IG_stress_	3.60 (1.88)	4.03 (2.03)	*	0.230 (0.01–0.42)	0.022	3.89 (2.01)	4.67 (1.88)	*	0.399 (0.06–0.73)	0.071
CG	5.23 (1.93)	5.37 (1.89)		0.074 (−0.01–0.16)						
*Cognitive abilities*										
Mental speed										
IG_mult_	2.38 (0.46)	2.56 (0.47)	***	0.386 (0.31–0.46)		2.42 (0.45)	2.65 (0.44)	***	0.508 (0.41–0.61)	
IG_TTT_	2.27 (0.49)	2.51 (0.49)	***	0.500 (0.34–0.66)	0.056	2.25 (0.54)	2.54 (0.49)	***	0.546 (0.28–0.81)	0.018
IG_comp_	2.40 (0.40)	2.62 (0.36)	***	0.551 (0.37–0.73)	0.080	2.35 (0.39)	2.57 (0.36)	***	0.576 (0.31–0.85)	0.033
IG_cogn_	2.39 (0.45)	2.56 (0.47)	***	0.388 (0.24–0.54)	0.001	2.43 (0.45)	2.64 (0.41)	***	0.492 (0.29–0.70)	0.008
IG_stress_	2.20 (0.47)	2.43 (0.42)	***	0.502 (0.29–0.72)	0.057	2.20 (0.38)	2.49 (0.36)	***	0.775 (0.44–1.11)	0.127
CG	2.39 (0.45)	2.43 (0.47)	*	0.096 (0.01–0.19)						
Concentration										
IG_mult_	60.54 (14.29)	68.02 (15.36)	***	0.503 (0.38–0.62)		61.42 (14.31)	72.33 (14.26)	***	0.764 (0.60–0.93)	
IG_TTT_	58.11 (13.96)	67.60 (14.95)	***	0.654 (0.40–0.91)	0.073	57.85 (13.49)	69.62 (12.34)	***	0.906 (0.54–1.27)	0.066
IG_comp_	66.78 (11.24)	70.72 (11.39)	**	0.349 (0.12–0.58)	0.075	64.09 (11.30)	67.74 (11.39)	*	0.322 (0.04–0.61)	0.091
IG_cogn_	59.70 (13.01)	64.83 (12.28)	**	0.405 (0.15–0.66)	0.048	60.98 (14.00)	66.65 (13.75)	***	0.408 (0.18–0.64)	0.171
IG_stress_	58.57 (14.10)	63.06 (14.21)	**	0.317 (0.11–0.52)	0.091	59.32 (15.46)	66.46 (15.71)	*	0.457 (0.14–0.78)	0.147
CG	60.54 (14.29)	68.02 (15.36)	***	0.373 (0.24–0.50)						

*p < .05; **p < .01; ***p < .001

Concerning self-concept of professional competence, in IG_mult_ scores in expertise, methodological competencies and affective commitment improved significantly compared to CG. For flexibility significance was missed, however, with near to small effect in favour of IG_mult_ (see Table 3). In addition, while *t*-tests showed that expertise was rated lower in IG_mult_ than in CG at t1 (*p* = 0.008, *d* = 0.253), there was no significant difference at t2 (*p* = 0.847). In methodological competencies no significant difference resulted comparing IG_mult_ and CG at t1 (*p* = 0.382), but they were rated significantly higher in IG_mult_ than in CG at t2 (*p* = 0.008, *d* = 0.256).

Regarding coping with stress, scores in willingness to work to exhaustion, distancing ability and inner calm changed significantly in IG_mult_ compared to CG. For resignation tendency significance was missed, but there was a near to small effect with greater improvement in IG_mult_ (see Table 3). *t*-tests revealed that at t1 in IG_mult_ inner calm was significantly lower (*p* = 0.001, *d* = 0.309) and resignation tendency significantly higher (*p* = 0.008, *d* = 0.255) than in CG. After training, these differences were noticeably reduced.

For cognitive abilities significant interactions resulted between time and group for mental speed (medium effect) and concentration in favour of IG_mult_ (see Table 3). In addition, at t1 mental speed did not differ significantly between IG_mult_ and CG (*p* = 0.916), whereas IG_mult_ outperformed CG at t2 (*p* = 0.004, *d* = 0.278). Concentration was descriptively lower in IG_mult_ than in CG at t1 and higher at t2.

Table 4 displays results of *t*-tests comparing intra-group developments from t1 to t2 (t1/t2) and t1 to t3 (t1/t3) for those variables in which development of IG_mult_ had shown to be significantly more positive than in CG. Table 4 also includes comparisons of effect sizes (Cohen’s *q*) between IG_mult_ and IG_TTT_, IG_comp_, IG_cogn_, IG_stress_ for both intervals. Effects of significant results were small, unless stated otherwise.

Subjective health improved significantly t1/t2 both in IG_mult_ and IG_TTT_. There was a small long-term effect in IG_TTT_, but significance was missed; effect size was significantly larger than in IG_mult_. For all other groups, there were no significant differences t1/t2, respectively, t1/t3, for subjective health. In IG_cogn_ the difference t1/t3 was not significant, but there was a small effect significantly larger than in IG_mult_.

Self-efficacy was significantly higher at t2 and t3 than at t1 in IG_mult_. For IG_TTT_, also significant differences resulted t1/t2, respectively, t1/t3 (medium effect). The effect t1/t2 was comparable for IG_TTT_ and IG_mult_; the effect t1/t3 was significantly larger in IG_TTT_. For all other groups, there were no significant improvements t1/t2 and t1/t3.

Concerning self-concept of professional competence, IG_mult_ also improved significantly t1/t2 and t1/t3 in expertise and t1/t2 in methodological competencies. While affective commitment improved descriptively in IG_mult_ t1/t2, there was a significant decrease in CG. For IG_TTT_ there were significant improvements t1/t2 for expertise and flexibility. All effects for IG_TTT_ did not differ significantly from IG_mult_. IG_comp_ improved significantly t1/t2 in expertise, methodological competencies and flexibility. There were also significant long-term effects for the first two variables and a small effect for flexibility, but here significance was missed. Effects did not differ significantly from IG_mult_.

Regarding coping with stress, no significant differences resulted within CG t1/t2. But both in IG_mult_ and IG_TTT_, t1/t2 and t1/t3 were significant for all four variables—distancing ability, resignation tendency, inner calm and willingness to work to exhaustion—except for the latter in IG_TTT_ t1/t3. There was a small effect, but significance was missed. The other effect sizes did not differ significantly from IG_mult_, except for resignation tendency. In IG_TTT_ the decrease in resignation tendency t1/t2 had a medium effect. In IG_comp_ there were significant improvements t1/t2 for resignation tendency and inner calm, but no significant long-term effects. The corresponding effect sizes did not differ significantly from IG_mult_. In IG_stress_ there were significant differences t1/t2 for distancing ability, resignation tendency and inner calm. For willingness to work to exhaustion there was a small effect, but significance was missed. Except for distancing ability there also were significant long-term effects. Effect sizes did not differ significantly from IG_mult,_ except for the reduction in resignation tendency t1/t2; the effect size was significantly larger in IG_stress_ than in IG_mult_.

For all intervention groups changes in both cognitive variables t1/t2, respectively, t1/t3, were significant with small to large effect sizes. Effect sizes in IG_TTT_ did not differ significantly from IG_mult_. Effect sizes t1/t2 in single-component versions also did not differ significantly from IG_mult_. But for t1/t3, the effect size for mental speed was significantly higher in IG_stress_ than in IG_mult_; the effect size for concentration was significantly higher in IG_mult_ than in IG_stress_ and IG_cogn_. The remaining effects did not differ significantly from IG_mult_.

## Discussion

This study aimed at examining the effects of an intervention for adults aged 50+. For five concepts under investigation—health, self-efficacy, self-concept of professional competence, coping with stress and cognitive abilities—participants of the multi-component training showed a significantly more favourable development over the time span of training than the control group. Short-term and long-term effects within the intervention group (research question 1) were found for self-efficacy, self-concept of professional competence, coping with stress and cognitive abilities; short-term effects were additionally obtained for subjective health. The long-term effects six months after training indicate that participants adopted self-reflection and implemented exercises and psychologically more adequate behaviour into their everyday lives. The psychomotoric exercises can easily be integrated into daily routines in varying difficulty, which is particularly important regarding cognitive advancements (O’Connell and Robertson 2012). Besides, dealing with new contents and thus perceiving oneself as a cognitively active person might have raised participants’ willingness to challenge their cognitive abilities, contributing to cognitive long-term effects (Jackson et al. 2012).

The intervention showed to be particularly effective regarding cognitive abilities. The fact that the tested tasks were not similar to the trained tasks is highly relevant as transfer from training to basic cognitive skills is not easily obtained (Simons et al. 2016), yet crucial for training benefits in everyday life. Concentration and mental speed are basic cognitive abilities relevant for a variety of cognitive processes, like higher cognitive functions, which are important in private and working life (e.g. problem solving; Vance et al. 2010). Training-based improvements in mental speed show transfer effects to daily actions and self-rated health in higher age (Wolinsky et al. 2010).

Regarding enhanced coping with stress, reduced willingness to work to exhaustion and resignation tendency as well as increased distancing ability and inner calm indicate health-enhancing behaviour in dealing with professional requirements. Distancing ability is highly relevant for sufficient recovery and thus for preventing recurring self-reinforcing strain (Hillert et al. 2012). The results coincide with findings showing effectiveness of stress management interventions in occupational settings (Richardson and Rothstein 2008). Also, self-efficacy was promoted, which is highly relevant in working contexts as it is associated with (a) constructive coping and is therefore discussed as protective resource regarding job strain, (b) developing healthy behaviour and (c) willingness to participate in further education and continuously developing professional skills (Schmid and Pfetsch 2018; Schwarzer and Hallum 2008).

In the light of changing demands in work contexts, promotion of supra-disciplinary skills like expertise, methodological competencies and flexibility gains importance. Training effects imply that independent and efficient handling of tasks, creativity in developing solutions as well as adaptability and tolerance of ambiguity were promoted (Hossiep and Paschen 2003). The effect found regarding affective commitment is mainly due to a decrease in the control group and can only partly be attributed to training; other influences, like characteristics of work tasks or leadership, are probably also important (Westphal and Gmür 2009).

As compared to the control group, training had no statistical effect on some variables included in the concepts of professional competence and coping with stress. However, there are plausible reasons for why these variables could have been—at the most—indirectly influenced by training: Some of the respective abilities were not explicitly trained in the interventions (e.g. proactive problem solving, social competencies); others are highly dependent on situational conditions or the general personal situation (e.g. experiences of success at work, life satisfaction, social support). Furthermore, the awareness of vocational competencies (like specialist skills, handling of work equipment) and social skills might have already been higher before training, as their daily use is more obvious or as they are often proven by certificates. Items measuring motivation aim partly at attitudes and behaviour contrary to successful stress management, like self-imposed excessive aspirations (Kaluza 2018). Subjective importance of work, professional ambition and striving for perfection are problematic, if they exceed a certain level. Therefore, relatively low, respectively, medium values might be most health-promoting and consistent with the training’s objectives.

Single-component trainings showed domain-specific effects as expected (research question 3). Additional effects were found for competence training regarding coping with stress, i.e. resignation tendency and inner calm. This can be explained by an increased perception of personal strength and resources. Surprisingly, cognitive effects were obtained in all single-component trainings. Cognitive abilities might have been promoted by the following characteristics, being part of all single-component trainings: (a) dealing intensively with new learning contents (Iizuka et al. 2019b), (b) psychomotoric exercises (Joubert and Chainay 2018; Oswald et al. 2006), (c) communication and social interaction (Iizuka et al. 2019b) and (d) metacognitive elements (O’Connell and Robertson 2012). Improved stress management is likely to contribute to cognitive improvement (Mache and Harth 2017), possibly by releasing cognitive resources (Hubbard and Byler 2016) or by reducing test anxiety and thus enhancing test performance (Knigge-Illner 2009). Besides, competence training might have strengthened self-confidence in cognitive abilities, contributing to challenging and thus promoting them (Chasteen et al. 2005; Jackson et al. 2012).

The effects of single-component trainings were overall comparable to those of the multi-component training. However, comparability of multi-component and single-component versions regarding cognitive improvements may be limited due to possible retest effects. These are likely to be more pronounced in the single-component trainings because of the shorter interval between tests, possibly resulting in an overestimation of cognitive effects (Salthouse 2009). Further evaluations within the scope of the project also indicated a stronger influence of retest effects in single-component trainings (Stahlhofen 2021). To counteract this problem in future studies, tests with parallel versions should be administered. An alternative explanation for similar cognitive improvements could be that a training of 7 weeks was sufficient to evoke equivalent effects to an intervention of 15 weeks.

The results indicate that multi-component training has not necessarily larger effects, but a broader impact than single-component trainings (research question 4). Particularly, subjective health and self-efficacy were only promoted by the multi-component version. Possible explanations are the interaction of different training modules enhancing additional effects and the prolonged time span of training and mentoring. Specific mechanisms underlying these synergy effects will have to be subject to further research. The results conform to findings showing advantages of combined trainings for improvement of subjective health ratings and of diverse and combined cognitive, metacognitive and psychomotoric exercises for maintaining and promoting abilities in mature adults (O’Connell and Robertson 2012; Oswald et al. 2006).

The results further show that courses administered by trained instructors and the programme’s designers were generally equally effective, indicating that training effects are independent of instructors (research question 2). These findings coincide with research identifying TTT-programmes as an effective educational approach (Pearce et al. 2012); they contradict views indicating that programmes are less effective when not administered by the programme’s designers (Beelmann and Karing 2014).

Extensive evaluation of training effectiveness using different methodological approaches is a strength of this study. Nevertheless, methodological limitations must be considered. The multi-component training does not only differ from the single-component trainings regarding its structure, but also regarding the total training time. However, this problem always emerges when evaluating a holistic training based on a comparison with the effects of its single components. If the trainings’ contents are to be comparable, a difference in total time is unavoidable. Also, companies and participants took part voluntarily, participants were not randomly assigned to groups and assessors were not blind to participant group allocation due to organizational reasons. It cannot be precluded that participating persons and companies systematically differ from those not participating. For example, the sample had a rather high educational level for this age cohort, reflecting higher willingness to pursue education found in adults with higher school qualifications (Bundesministerium für Bildung und Forschung 2021). Differences between intervention and control groups before training indicate self-selection and can be explained by the motivation to participate in a training programme. Training closed the gap between these groups and, thus, addressed the target group. Generalizability of results is limited to well-educated persons taking part voluntarily in further education and completing the programme, reflecting high interest and intrinsic motivation. Furthermore, relatively small sample sizes of single-component and TTT-trainings must be considered, because they impede detection of statistically significant differences, especially regarding long-term effects. Post hoc power analyses indicate that larger samples would indeed lead to significance in variables for which small effects were found in this study. Further research should focus on evaluating interventions with optimised methodical means to confirm the results. To control for placebo-effects and effects of receiving special attention by trainers on outcome measures, an additional active control group receiving a different intervention should be included. In this study, unfortunately, none of the participating companies agreed to release their employees during work hours for such a control group. Besides, it seems worthwhile investigating training effectiveness when administered by trainers in business practice using the manual without having attended the TTT-programme.

This intervention study presents a well-evaluated, field-proven training programme designed for mature employees. The training’s high acceptance by the target group, providing a low-threshold offer and the evaluation of trainings administered by trained instructors are strengths of the programme regarding practical implementation. To ensure training effectiveness in business practice, psychological background knowledge and ideally practical training experience are important as well as knowledge about the training, its implementation and usage of manuals and training material (Beelmann and Karing 2014). Furthermore, a combination with structural organizational measures should be standard practice to allow working and learning under ageing-appropriate and health-promoting conditions and to ensure lasting effects of health promotion programmes (Bieling et al. 2015).

## Data Availability

Not applicable.
